# How Did Governments Address the Needs of People With Disabilities During the COVID-19 Pandemic? An Analysis of 14 Countries’ Policies Based on the UN Convention on the Rights of Persons With Disabilities

**DOI:** 10.34172/ijhpm.2023.7111

**Published:** 2023-05-17

**Authors:** Keiko Shikako, Raphael Lencucha, Matthew Hunt, Sébastien Jodoin, Mayada Elsabbagh, Anne Hudon, Derrick Cogburn, Ananya Chandra, Anna Gignac-Eddy, Nilani Ananthamoorthy, Rachel Martens

**Affiliations:** ^1^School of Physical and Occupational Therapy, McGill University, Montreal, QC, Canada; ^2^Center for Interdisciplinaire Research in Rehabilitation of the Greater Montreal (CRIR), Montreal, QC, Canada; ^3^Faculty of Law, McGill University, Montreal, QC, Canada; ^4^Montreal Neurological Institute, McGill University, Montreal, QC, Canada; ^5^School of Rehabiltiation, University of Montreal, Montreal, QC, Canada; ^6^School of International Service and Kogod School of Business, American University, Washington, DC, USA; ^7^Institute on Disability and Public Policy (IDPP), American University, Washington, DC, USA; ^8^Kids Brain Health Network, CanChild, Calgary, AB, Canada

**Keywords:** Disability, Human Rights, COVID-19, Policy

## Abstract

**Background:** People with disabilities have experienced heightened social risks in the context of the pandemic, resulting in higher rates of infection and mortality. They have also borne elevated burdens associated with public health measures. The United Nations Convention on the Rights of Persons with Disabilities (UNCRPD) obliges its 184 state parties to eliminate discrimination and ensure equality and inclusion for persons with disabilities, including protection and safety in situations of emergency. It remains unclear to what extent national COVID-19 policies have aligned with these commitments under the UNCRPD. Our objective in this exploratory study was to assess alignment between the UNCRPD indicators and COVID-19 policies from 14 countries with the goal of informing policy development that is inclusive of persons with disabilities and responsive to rights under the UNCRPD.

**Methods:** We identified COVID-19 policy documents from 14 purposively selected countries. Country selection considered diversity based on geographic regions and national income levels, with restriction to those countries that had ratified the UNCRPD and had English or French as an official language. We used a computational text mining approach and developed a complex multilevel dictionary or categorization model based on the UNCRPD Bridging the Gap indicators proposed by the Office of the High Commissioner on Human Rights (OHCHR). This dictionary was used to assess the extent to which indicators across the entirety of the UNCRPD were represented in the selected policies. We analyzed frequency of associations with UNCRPD, as well as conducting ‘key word in context’ analyses to identify themes.

**Results:** We identified 764 COVID-19 national policy documents from the period of January 2020 to June 2021. When analyzed in relation to the Articles of the UNCRPD, the most frequently identified were Articles 11 (risk and humanitarian emergencies), 23 (home and family), 24 (education), and 19 (community living). Six countries produced 27 policies that were specifically focused on disability. Common themes within these documents included continuation of services, intersectionality and equity, and disability considerations in regulations and public health measures.

**Conclusion:** Analyzing country policies in light of the UNCRPD offers important insights about how these policies do and do not align with states’ commitments. As new policies are developed and existing ones revised, more comprehensive approaches to addressing the rights of persons with disabilities are urgently needed.

## Background

**Key Messages**

**Implications for policy makers**

- The variability of policy measures across countries suggests that more work needs to be done to harmonize effective and equitable policy responses for persons with disability during a pandemic or emergency context.
- While policies consistently articulated public health measures to protect the physical health of populations as a whole, there remains a need to expand the scope of policy to address the needs of daily life including social contact, mental health, financial and other supports, and access to education, employment, amongst others, especially for members of the disability community.
- The United Nations Convention on the Rights of Persons with Disabilities (UNCRPD) provides an important normative reference point to establish comprehensive policy responses and monitor policy content to ensure that the needs of persons with disabilities are met.

**Implications for the public**

The countries reviewed in this project identified that persons with disabilities were at risk and required particular attention during the pandemic, mostly focusing on accessibility of testing centers and disease prevention measures for people living in group homes with limited mention of the intersecting needs associated with social interaction, mental health, education and others. Persons with disability can use the United Nations Convention on the Rights of Persons with Disabilities (UNCRPD) as a reference for assessing the policy responses during emergencies and request access to necessary services and supports. It is important that the public consider the needs of persons with disability and support this community and organizations of persons with disabilities and service providers across sectors (education, health, leisure, community living) to ensure that emergency preparedness addresses the provisions of the UNCRPD.

Effective pandemic response by governments requires comprehensive assessment of the needs of their citizens, which may vary due to factors such as gender, ethnicity, socio-economic status, or disability.^1^ The COVID-19 pandemic and related control measures increased the health and social risks for many persons with disabilities.^2,3^ The United Nations Convention on the Rights of Persons with Disabilities (UNCRPD) outlines provisions for the protection and promotion of the civil, political, social, economic, and cultural rights of persons with disabilities. Under pandemic conditions, the requirement of governments to identify and respond to the needs of persons with disabilities is amplified by the new or exacerbated vulnerabilities for these individuals, and the need to prioritize actions and resources across the entire population.^4^

 People with chronic health conditions have experienced higher rates of infection and mortality from COVID-19 across different regions of the world.^5^ Moreover, institutionalization has been a factor significantly increasing the risk of becoming infected with, and dying from, COVID-19.^6-8^ Analysis of international data shows that persons with intellectual and developmental disabilities were more likely to be infected with COVID-19 and more likely to die from it than others.^9-11^ During the pandemic, persons with disabilities have experienced barriers to access public health information, difficulties complying with social distancing orders due to support needs or because of institutional housing, and challenges pertaining to personal protective measures, such as handwashing due to inaccessible sinks or hand pumps.^10,12,13^ A rapid literature review published during the COVID-19 pandemic found that persons with physical disabilities experienced decreased access to healthcare, including essential rehabilitation services, exacerbated mental health challenges, and community participation restrictions.^14^ Children with disabilities have struggled to access essential health and education services during the pandemic, and parents have encountered barriers to accessing respite and support services to guarantee continuity of care.^10,15^ This situation of heightened and unique vulnerability led to calls for disability inclusive pandemic and health emergency measures that include attention to different communication needs, access to health and educational services and supports, and disability rights training for healthcare workers.^2^

 Many concerns have been expressed regarding government policy responses to the needs of persons with disabilities. For example, in several US States, lawsuits were launched about discrimination and neglect found in healthcare triage protocols and overall logics of resource allocation.^16^ Impacts have also been felt at the level of programs. In one Canadian province, access to assistive devices was impeded when, at the beginning of the pandemic, assistive device programs were categorized as a non-essential service and faced restrictions in their operations.^17^ A qualitative analysis into rehabilitative services in the Gauteng province of South Africa found that lockdown measures impeded the quality of care, the ability of service-users to access that care, and the mental wellbeing of service providers, noting that vulnerable populations facing socioeconomic disparities were most affected.^18^ In India, a survey conducted by the National Centre for Promotion of Employment for Disabled People found that 73% of interviewees faced challenges specifically due to the lockdown including but not limited to financial struggles, food insecurity, and barriers in access to healthcare.^19^

 The UNCPRD obliges its 184 state parties to take measures to eliminate discrimination and ensure equality and inclusion for persons with disabilities across a range of fields, including healthcare, education, employment and transportation, amongst others — and that may be particularly important to guide government responses during an emergency such a pandemic.^20-22^ In fact, Article 11 of the UNCRPD specifically requires that states adopt measures to ensure the protection and safety of persons with disabilities in situations of risk. However, the extent to which national policies are aligned with commitments under the UNCRPD remains unclear.

 During the COVID-19 pandemic, the value of monitoring, sharing and learning from experiences across countries has been clearly demonstrated.^23^ Cross-country comparison of COVID-19–control policies, for instance, have allowed for a better understanding of the effectiveness of different policies in reducing the spread of the virus.^24^ Collecting and analyzing national level policies can support understanding of how different countries have attended to the needs of persons with disabilities, and to draw comparisons and lessons from one country to the other. Following this logic, we used a novel methodology to conduct a comprehensive international analysis of a purposeful stratified selection (or sample) of the national policies of 14 countries in response to the COVID-19 outbreak.

 The objective of this study was to analyse countries’ public policies during the COVID-19 pandemic to assess their alignment with the obligations of states under the UNCRPD. Our aim in undertaking this analysis is to inform and support the development of policy responses that are inclusive of persons with disabilities during public health emergencies and beyond.

## Methods

 Our analysis took a computational text mining approach. We were guided by the Cross-Industry Standard Process for Data Mining approach for text mining which guides researchers through six stages of problem formulation, data collection and preparation, model development, analysis, and deployment.^25^ As a core part of this approach, we developed a complex multilevel dictionary or categorization model based on the UNCRPD Bridging the Gap indicators proposed by the Office of the High Commissioner on Human Rights (OHCHR).^26^ This dictionary was used to assess the extent to which indicators across the entirety of UNCRPD were represented in the selected country policies.

###  Country Selection

 We selected a purposeful sample of countries according to four key criteria that were deemed important to respond to the research question: Our first criterion involved ratification of the UNCRPD. We further stratified the sample by those countries that had French or English as an official language. Our third criterion was income. We purposively selected a representative sample of countries across four income categories (low, low-middle, upper-middle, and high) based on World Bank classifications and across each of the United Nations geographic regions. Based on these selection criteria, the final list of countries included Australia, Canada, Fiji, France, Guinea, Haiti, India, Ireland, Jamaica, Malawi, Philippines, Rwanda, South Africa, and Zimbabwe. At the start of this study, Haiti was classified as a low-income country by the World Bank Lending Group. It has since been reclassified as a lower-middle income country, however for the purposes of this study, Haiti was considered in the low-income category.

###  Policy Documents Search and Selection

**Figure 1:**
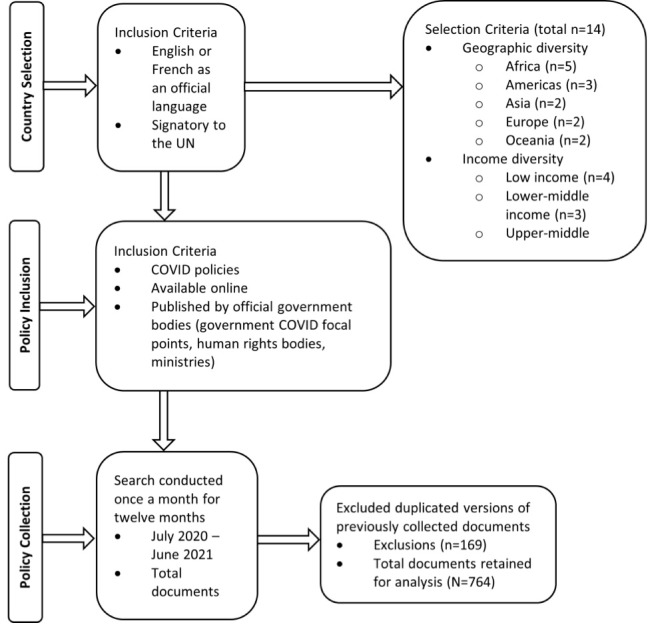
Selection Criteria and Policy Documents Review Flow. Abbreviation: UN, United Nations.

 Beginning in July 2020, we carried out a monthly collection of policy documents related to COVID-19 from the government websites or focal COVID-19 platforms of selected countries. The final data collection took place in June 2021. We used the following inclusion criteria: (1) national-level documents accessible online and published by national authorities (ie, government), (2) in English or French, and (3) specific to COVID-19. Documents were excluded if they were social media postings, previously published policies related to other infectious disease outbreaks or pandemics not specific to COVID-19, or sub-national policies. In countries where we identified few eligible documents, we supplemented our data collection by searching sources such as the COVID-19 Law Lab,^27^ AfricanLii,^28^ and Asian Preparedness Partnership.^29^ We also accessed Official Government Gazettes that were not part of the government website, but official state sources for decrees, policy documents, etc for the Philippines, Zimbabwe, Rwanda, and France. Figure 1 illustrates the countries (cases) selection criteria and policy documents selection process (Please refer to Supplementary file 1 for a complete list of document sources and detailed search strategy).

###  Categorization Models

**Figure 2:**
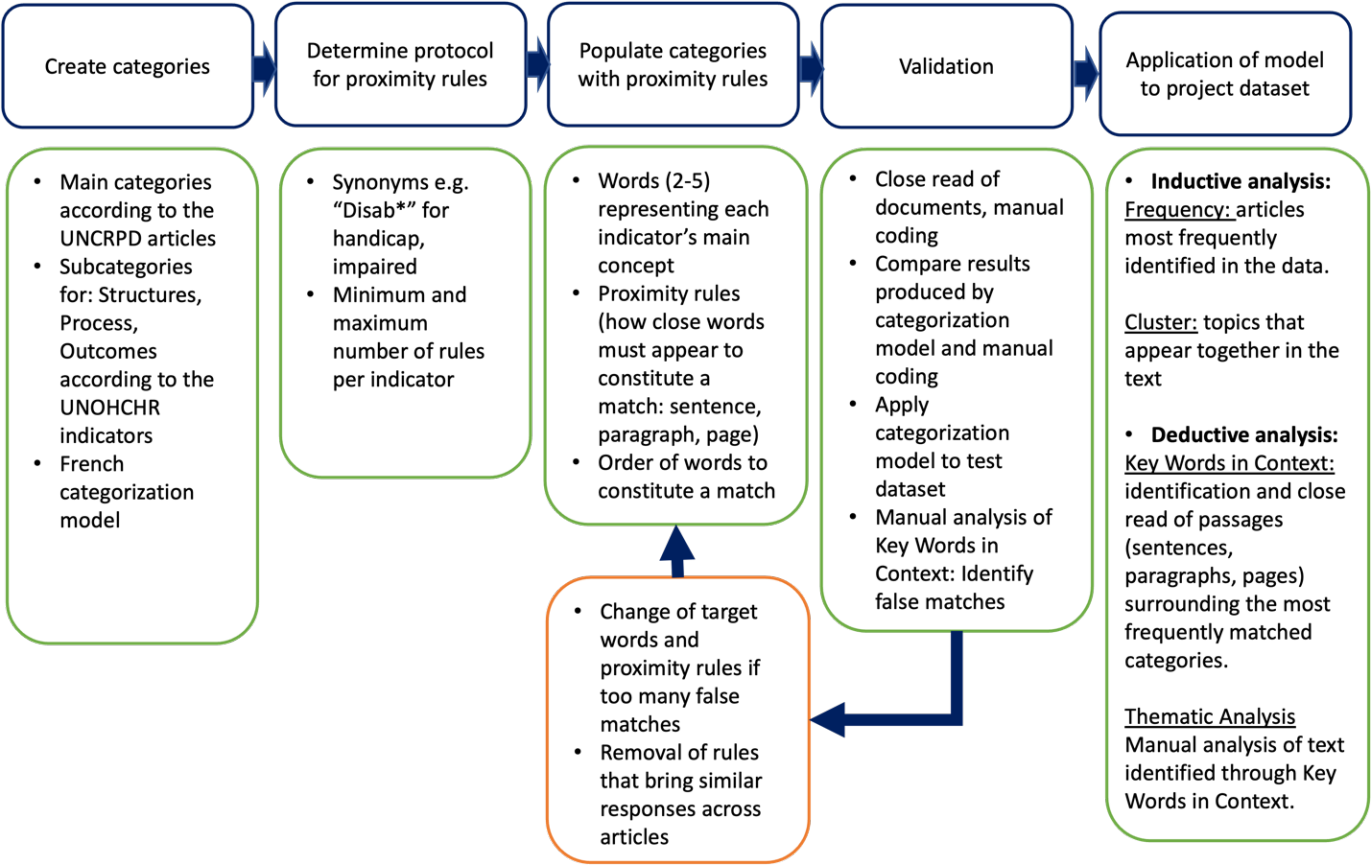
Summary Description of Categorization Model Development. Abbreviations: UNCRPD, United Nations Convention on the Rights of Persons with Disabilities; UNOHCHR, United Nations Office for the High Commissioner in Human Rights.

 Our text mining took a “bag of words” approach, rather than natural language processing. This statistical text mining approach allows us to convert our text into a corpus that numerically represents all the words in all the selected policy documents.^30^ To assess the extent to which the UNCRPD indicators were present or absent in the policy documents, we built a multilevel categorization model, also known as a dictionary, representing these indicators which include all 50 Articles of the UNCRPD (see Figure 2). Each Article in the UNCRPD was given a separate category in the dictionary and then subcategorized into “Structure,” “Process,” and “Outcomes.” Next, each subcategory was further organized into attributes that were specific to that Article’s indicators. For example, indicators for Article 6 (Women and Girls with Disabilities) are organized under the attributes: “Non-discrimination and Equality” and “Full Development, Advancement, and Empowerment of Women.” These were each given a subtopic under each of the three subcategories (Structures, Process, Outcomes) (see examples in Supplementary file 2). The categorization model is more than a key word search. It is a complex multi-layer algorithm that includes a combination of words and concepts that are translated into sentences by the group of researchers, which includes several disability and policy scholars and advocates (to ascertain the concepts were represented in the algorithm). After the concepts were translated into sentences, the categorization model was initially piloted using two different data sets (applied into the datasets and the results verified manually by the group). When disparities were encountered (eg, a category would bring results that were not related to the concept), the model was further adjusted and the categories re-coded and tested again, until the text mining results were satisfactorily equivalent to the “human” analysis. Then, this model was tested with this dataset and adjustments were done if any disparities were still identified, until we could reliably obtain responses from the analysis that were representative of what a human analysis would capture in the text. Finally, each indicator was categorized into subcategories and given anywhere between one and six proximity rules (the central word conveying the context of the Article, and the line, paragraph, document proximity) to search the data for phrases and sentences that reference the content of that indicator. The proximity rules function by using anywhere between two to five words or phrases that are coded such that if they are found in a particular pattern or in proximity to one another then the program would save that sentence as a reference to the category or subcategory. At the subcategory level, between five to twenty rules were created based on the number of indicators in that category as well as their complexity. All rules contained “disability” (or disab*) as a proximity rule, meaning we captured the content of each Article in relation to disabilities in the text.

###  Data Extraction

 Each document was categorized according to the following characteristics: Country, Region, Income category, Language, Specificity (ie, whether entire document is focused on disability or if the document’s scope is wider), and the Source (eg, federal government, disability ministry) through which it was identified. The documents were uploaded with these variables to WordStat, a commercial text mining software package, for analysis. These variables allowed the team to incorporate the country context and make initial comparisons across variables. To further contextualize the analysis, we obtained COVID-19-related information (eg, number of cases, stringency measures) for each country from the Our World in Data database.^31^

###  Data Analysis

Figure 2 illustrates the process to develop the categorization model and the analysis. The first search was conducted in July 2020 and collected documents from the period January to July 2020. New documents were then collected once a month until June 2021. We analyzed the documents using both an inductive (exploratory) and deductive (confirmatory) text mining approach. The inductive analysis helped us to explore and better understand our dataset. It began with keyword and phrase frequency analysis, and progressed to other inductive techniques such as cluster analysis. Our word and phrase frequency analysis also used a technique called “term frequency by inverted document frequency.” This technique is a heuristic that allows the researcher using the bag of words approach to identify “important” words that appear frequently in a dataset but reduces the impact of those words or phrases that appear too frequently. These automated processes of identifying the frequency of words, phrases, and topics allowed us to detect thematic clusters in the data corpus. The deductive analysis was conducted using the categorization model created by the research team to identify the frequency that UNCRPD Articles were cited across documents. We also used the “Key Words in Context (KWIC)” function of WordStat to validate our dictionary and to conduct a content analysis of the policies. This function allowed our team to situate the findings from the deductive analysis (ie, alignment with the UNCRPD Articles) within the document context (sentence, paragraph, document).

## Results

 A total of 1037 documents were retrieved for analysis. After removing duplicates, a total of 764 documents were analyzed. Here we describe the distribution of policies across countries and income categories, followed by our inductive analysis and deductive analysis which highlights the alignment of content associated with the Articles and indicators of the UNCRPD.

###  COVID-19 Policies Per Country

**Figure 3:**
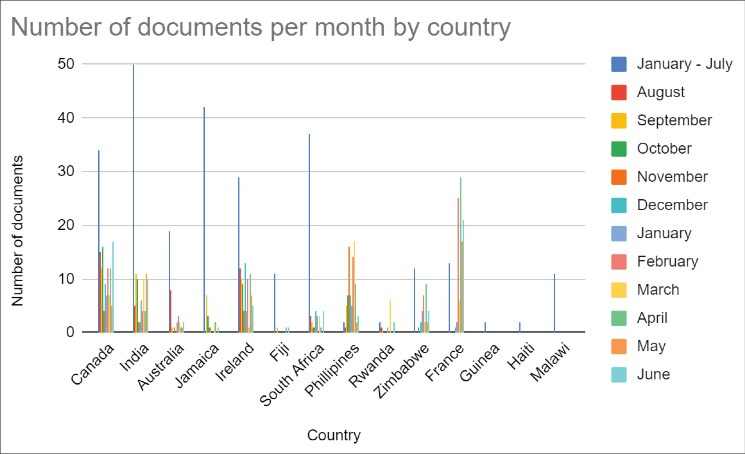
Number of Documents Collected Per Month for Each of the 14 Countries From July 2020 to June 2021.

 The bulk of COVID-19-related policies were established by national governments at the beginning of the outbreak between January and July 2020, with 32% of the collected documents being released during that period. The largest absolute (ie, not adjusted for population) number of policies were established by France (n = 146), followed by India (n = 120), Canada (n = 82), and the Philippines (n = 79) (See Figure 3 for volume of documents retrieved by country during the data collection period). The distribution of total number of policies between January and July 2020 was broadly similar across countries from high (n = 85 documents/4 countries; average of 21.5 documents/country), upper-middle (n = 85 documents/3 countries; average of 28.3 documents/country), and lower-middle (n = 61 documents/4 countries; average of 15.3 documents/country) income categories. However, considerably fewer policies were identified among low-income countries (n = 16 documents/3 countries; average of 5.3 documents/country) relative to the other income categories. It should be noted that the countries we included in our analysis within the low-income category generally experienced their first waves of COVID-19 later than other nations included in the analysis,^32^ which could explain the lower number of documents produced by these governments. It should also be noted that the number of documents is not an indication of the quality or scope of the government policy.

###  Inductive Analysis: Word and Topic Frequencies 

####  Topics Identified

 The inductive analysis identified the most frequent phrases (Supplementary file 3) and topics (Supplementary file 4) contained in policy documents. The inductive analysis serves to describe the dataset, and verify the general content of the documents retrieved (ie, validating the content as COVID-19 specific documents) before we looked specifically into the disability-related content. Public Health was the most frequently mentioned phrase. It was mentioned in 44.7% of 332 documents. Health Services was the most coherent topic (identified as a topic in the greatest number of documents), identified in 86% of 645 documents. Amongst the most frequent phrases and topics, most pertained to public health measures, such as physical distancing, hand hygiene and infection prevention and control. Mental health was the only health condition figuring among the most frequent phrases, and children and youth was the only population group identified in the topic analysis. This analysis seems to align with the COVID-19 prevention and containment focus of policies adopted during the first phase of the pandemic for which documents were collected.

 The cluster analysis (Supplementary file 5) assembles topics that are frequently mentioned together in the corpus of data to identify patterns of co-occurrence. We identified clusters addressing access to food supplies and essential services and public health prevention and control. Mentions of community settings co-occurred with potential for transmission and mitigation strategies.

###  Deductive Analysis: UNCRPD Categorization Model

####  General Frequencies 

**Figure 4:**
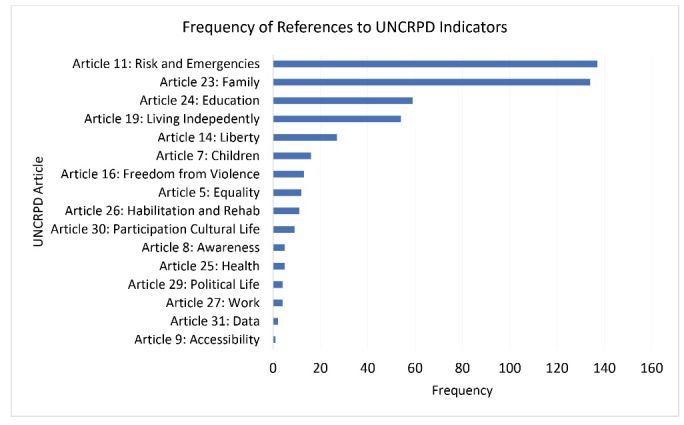
Frequency of References to UNCRPD Indicators. Number of times each Article of the UNCRPD was referenced in all documents (excluding duplicate or republished documents) for all 14 countries over the 12-month collection period. Abbreviation: UNCRPD, United Nations Convention on the Rights of Persons with Disabilities.

 Policy documents were analyzed with the UNCRPD categorization model to identify content most frequently associated with specific Articles. Not surprisingly, Article 11 (risk and humanitarian emergencies — associated with disability) had most content associated with in the totality of documents — it was identified 137 times in 62 policy documents (ie, cases) from 9 of 14 countries. The next most frequently identified content was associated with Article 23 (Respect for home and family). This content was derived from half the number of policy documents as Article 11 (n = 34), and from 11 of 14 countries. Third most common was Article 24 (Education) with 24 documents from 8 countries, while Article 19 (Community living) was fourth with linked content in 15 documents from 4 countries. The list of all the identified CRPD Articles with its respective frequencies can be found in Figure 4.

####  Frequencies Based on Income Categories

**Figure 5:**
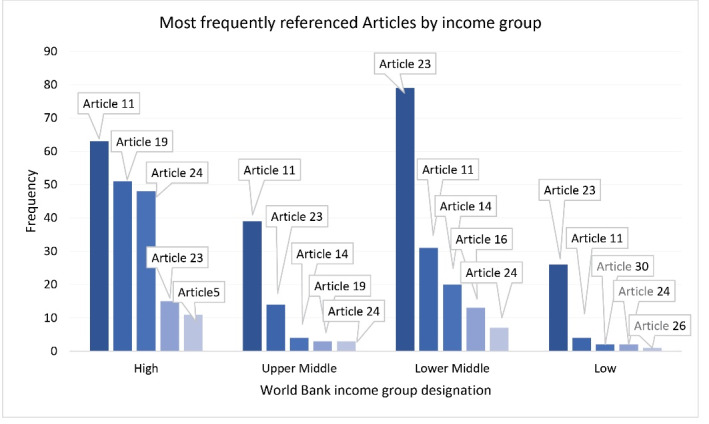
UNCRPD Articles Frequencies Per Countries’ Income Category Clusters. Note: Article 5 (Equality and Non-discrimination); Article 11 (Situations of Risk and Humanitarian Emergencies); Article 14 (Liberty and Security of person); Article 16 (Freedom from exploitation, violence, and abuse); Article 19 (Living Independently and Being included in the community); Article 23 (Respect for the home and family); Article 24 (Education); Article 26 (Habilitation and Rehabilitation); Article 27 (Work and Employment); Article 30 (Participation in Cultural Life, Recreation, Leisure, and Sport). Abbreviation: UNCRPD, United Nations Convention on the Rights of Persons with Disabilities.

 We analysed the frequency that specific UNCRPD Articles were captured according to the countries’ income categories to identify if there were any differences in the emphasis of one area over another across different income categories. Figure 5 illustrates the Articles’ frequencies per countries’ income category clusters. Articles 11 (Risk and Humanitarian Emergencies), 23 (Family), 19 (Community Living) and 24 (Education) remain the most captured Articles by our model. These Articles are found in the top five Articles for countries in our sample from three of the four income categories, except for the low-income and lower-middle-income categories where Articles 23, 11, and 24 are found to be within the top five most frequently identified Articles, but not 19. Several other Articles are found in the top five Articles within specific income categories. For the low-income category, Article 30 (Participation) was the third most commonly identified, and 26 (Rehabilitation) was the fifth. For the low middle-income category, Article 14 (Liberty and security) was third and Article 16 (Freedom from exploitation) was fourth, and for the upper middle category, Article 14 was third. Article 5 (Equality and Non-discrimination) was unique to the high-income category where it was the fifth most frequently identified Article.

####  Disability-Specific Policy

 Six countries (out of 14) in our sample produced policies focused solely on the needs of persons with disabilities: Australia Canada, France, India, Jamaica, Philippines, and South Africa. These six countries collectively produced 27 disability-specific documents.

**Figure 6:**
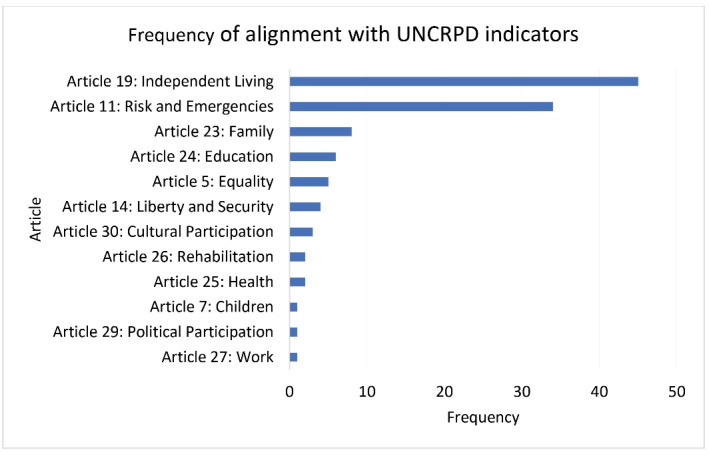
Frequency of Articles of the UNCRPD Captured in Policy Documents Pertaining Specifically to Persons With Disabilities (Excluding Duplicate or Republished Documents) for Six Countries That Published Disability-Specific Guidance. Abbreviation: UNCRPD, United Nations Convention on the Rights of Persons with Disabilities.

 We conducted a frequency analysis of the UNCRPD Articles, as well as a close read of the keywords in context on the most frequent categories in these disability-specific documents. Analysis of these documents most frequently identified content associated with Article 19 (Community living), content linked to Article 11 was captured in 8 of these 27 documents, associated with specific issues related to disability in the pandemic. Respect for the family (Article 23) was the third most frequently identified Article in these documents. The Right to Education (Article 24) was articulated in only two documents. Aspects related to the Right to Health (Article 25) were only identified in two documents as well. Figure 6 presents the frequency of items captured through our CRPD indicators model in the disability specific documents.

####  Key Words in Context Related to the Top Five Most Cited Articles From Disability-Specific Policies

 The KWIC analysis provided insights into the topics discussed in the policy documents as they relate to the associated Article of the UNCRPD. Here we present three dominant themes that cut across the top five Articles mentioned in the policy documents (see Table for themes and illustrative quotes).

**Table:** Quotes From Documents Illustrating the top 5 Most Frequent UNCRPD Articles

**Article**	**Theme**	**Quotes**	**Country**
Article 11 Risk and Humanitarian Emergencies	Continuation of services	“Continued access and uptake of welfare provisions and or emergency supports. Government agencies can aid the situation by pro-actively preserving and delivering benefits access in such a situation, such as advance payments of pensions and ensuring quick access to services. Continued service delivery may have beneficial impacts on reduction of post disaster morbidity and disability. Re-evaluate certification processes for PwDs to allow for more online or video-based assessments, in order to preserve access. Systems should adapt to include measures for ad hoc/temporary certifications so as to ensure adequate financial and non-financial supports. This is in consonance with sections 8 and 24 of RPWD act. Carer health is also imperative and should be proactively monitored by mental health professionals.”	India
Article 19 Community Living	Intersectionality	“Contact details of social isolation and disruption of daily routines, particularly in congregated settings such as group homes: Providing emergency support to families who are caring for family members who have behaviours of concern that may pose a risk to the person with disability and/or family members. Liaising with Aboriginal and Torres Strait Islander advocacy groups and communities to ensure that their needs are met. Ensuring that people who have not yet transitioned to the NDIS and people with disability who do not qualify for the NDIS, but may be vulnerable to COVID-19, receive the services and the supports they need.”	Australia
Article 24 Education	Intersectionality	“It should be expected that some children/youth will wear NMMs in schools that have not adopted NMMs policies. Staff and volunteers should monitor for, and address, any discrimination or bullying associated with this practice (whether stigmatization is experienced by those who wear masks, and/or those who do not) and how this can amplify discrimination or bullying due to other factors such as differences in gender, ethnicity, or ability.”	Canada
Article 25 Health	Equity	“In addition, the allocation COVID-19 vaccines and prioritization COVID-19 immunization shall be anchored to the following principles: Human well-being: where health, social and economic security, human rights and civil liberties all citizens and individuals are protected and promoted. Equal respect: where all human beings are treated equally and their interests are considered with equal moral consideration. Ensure that vaccine prioritization takes into account vulnerabilities, risks and needs groups because of underlying societal, geographic or biomedical factors.”	Philippines
Article 23 Respect for Family	Disability considerations in regulations	“Policies and procedures for hospitals, long-term care homes, COVID-19 Assessment Centres, clinics, family practice, other medical facilities and any organization that provide healthcare and supportive services to those with disabilities should provide permission in their directives on the accompaniment of essential supports at all stages of care within the healthcare environment.”	Canada

Abbreviations: UNCRPD, United Nations Convention on the Rights of Persons with Disabilities; PwDs, Persons With Disabilities; NDIS, National Disability Insurance; NMMs, non-medical masks; RPWD, Rigs of Persons With Disabilities.

###  Continuation of Services

 With all services interruptions there were several considerations about how service disruptions would impact persons with disabilities with a focus on home, institutional, and respite care and school, community, and health services. Emphasis was placed on how disruptions could be mitigated through some type of intervention. There was an emphasis placed on providing financial supports for families, and those persons with disabilities living in non-family environments (eg, group homes or institutions). Another feature of the policies pertaining to continuation of services was captured under Article 23 (Family) and 19 (Community) specific to ways to facilitate the ability to make choices about activities in the community during periods of restriction. In two countries the emphasis on support services were presented in relation to the need to address loneliness and isolation (Canada, India). Aligned with the Right to Education articulated in Article 2, only two documents referred to the needs of children with disabilities during school closures. The remainder focused on the need to maintain school-based services.

###  Intersectionality and Equity

 The intersectional vulnerabilities and needs was identified as a theme across some of the Article categories. For example, specific guidance was provided by the government of Canada to address issues that persons with intellectual disabilities could experience, such as anxiety and stress during COVID-19 testing. Intersectional needs were often associated with community living (Article 19). Aspects of community living captured in our model included safety considerations and risk for abuse, as well as maltreatment or neglect for persons with disabilities during prolonged confinement periods, including woman and children (Australia, Canada), and persons with behavior issues (Australia). Intersectionality was also raised in relation to the specific needs of communities. For example, documents from Australia noted the need to open lines of communication with Aboriginal groups about their specific needs.

 Intersectionality and equity were also associated with Article 24 (Education) where specific considerations were afforded for children with disabilities. In one of the documents from Canada, there was recognition that children with disabilities may not be able to wear a face mask and should not be discriminated against for this reason (Canada).

 Finally, equity was addressed multiple times in the last data collection period, when a few governments (eg, Philippines) with particular emphasis on vaccines distribution. Considering it was only the initial discussions about vaccines that were captured in our data collection period, the mentions in the documents address prioritization of specific groups including persons with disabilities and their direct care workers. For example, the government of India provided specific guidance in relation to assessing risks and prioritizing vaccination of persons with disabilities.

###  Disability Considerations in Regulations and Public Health Measures

 This theme was associated with several Articles. The content associated with Article 19 (Community living), emphasized issues pertaining to access to testing facilities and treatment centers for persons with disabilities, as well as other aspects of independence in the community amidst restrictive public health measures. Content linked to Article 11 was captured in 8 of these 31 documents. Article 11 supports the understanding that persons with disabilities are to be considered a vulnerable group requiring special consideration in pandemic management. Only India and Canada referred to disability specific guidance (eg, UNCRPD), prior national disability policy, and/or international guidance (ie, Sendai Framework) in their national policies. Content addressing Article 11 included communications of precautionary measures and announcements (Canada, France), specialized support services (Canada, France, India), accessibility of hand sanitizers and sanitization of personal assistive devices (eg, canes and wheelchairs) (India), and the need to assess safety, risk, accessibility and additional support services in different facilities where groups of vulnerable populations, such as persons with disabilities, would be concentrated (Philippines). There was reference to precautions that should be implemented by care providers and by the persons under their care, and the need for appropriate training and provisions of equipment and other accessibility measures for care providers (Canada, Ireland).

 Including persons with disabilities in decision-making processes is mandated in Articles 4 and 29 and is a mechanism for ensuring inclusive emergency responses pursuant to Article 11. The establishment of consultative committees was only mentioned in the policies of two countries (France and Canada). Strengthening community supports was a mechanism used in France, where a ‘solidarity platform’ was created so that individuals with disabilities could indicate their needs and receive help from the community. A complaint mechanism was implemented in Canada towards the end of our data collection period to address the lack of vaccination of healthcare and support care workers dealing directly with persons with disabilities.

## Discussion

 This study mapped the COVID-19 national policy documents of 14 countries distributed across regions and income categories during the period of January 2020 to June 2021 to analyse countries’ public policies during the COVID-19 pandemic and to assess their alignment with the obligations of states under the UNCRPD. Our aim in undertaking this analysis is to inform and support the development of policy responses that are inclusive of persons with disabilities during public health emergencies and beyond.

 We identified that the volume of policies published varied across countries, as did the content specific to persons with disabilities within national policies. We found that the focus of policies is largely on emergency aspects and persons with disabilities are considered in broad guidance for physical accessibility in testing centers, for example. There was some limited consideration for access to health services that may not be considered essential for the population at large but may be essential for persons with disabilities (eg, rehabilitation services), and that, regardless of country socio-economic profile.

 Six of the 14 countries included in this study adopted policies specifically designed to meet the needs of persons with disabilities, though these were highly variable in their alignment with the UNCRPD. While some countries noted that those at greatest risk should receive special attention, for example in the Philippines it was noted that vaccine access should be prioritized for those with greater burden (referring to healthcare workers, including those who provide direct services for persons with disabilities at home, but not persons with disabilities specifically), this was not reflected across the 14 countries. To address different needs of persons with disabilities, some national policies in our analysis considered provisions to guarantee the access of persons with disabilities to essential services provided in the community such as testing centers and personal protective measures (eg, physical accessibility of buildings and to hand sanitizing, and distribution of masks or exemptions to mask wearing). However, we did not identify in the policy documents other disability considerations of public health responses and measures that should be part of national level governments mandates, such as accessible (ie, sign languages, braille, easy read versions, audio description) official communication done by governments in relation to development of the pandemic, restrictions, and public health measures. For instance, deaf, and hard of hearing people in the United States had less access to information about precautionary measures and instructions in case of having symptoms than hearing individuals.^33^ A Latin America report on persons with disabilities during the pandemic identified a majority of persons with disabilities having difficulty seeing (as per categories of disability recommended by the Washington group on disability data collection),^34^ and access to public health measure and information was also compromised for this group.^35^ Persons with disabilities who require direct support from formal or informal caregivers also faced restrictions in their ability to follow social distance guidelines. The consideration for those most vulnerable has been a topic of study during the pandemic,^36^ and disaggregated data collection on disability, as proposed in Article 31 of the UNCRPD, would allow for the development of targeted, effective and inclusive measures. With the right data about specific disability groups, regulations for disability can be established early on, and timely decisions about services and structures could be more responsive to the pressing needs of persons with disabilities.

 Considerations of intersectionality was a theme captured in our analysis, with mention of some more vulnerable groups within the group of persons with disabilities such as children, woman, elderly, and those in the intersectionality of social determinants of health such as persons with disabilities living in poverty, living in rural areas or reserves, ethical and language minorities, and indigenous. Other studies confirm that some country-level policies considered the heightened risks for specific groups of persons with disabilities, including persons living in segregated settings such as institutions and group homes,^1^ individuals with intellectual disabilities,^37^ and children and woman, amongst other groups. Persons with disabilities are constantly disregarded in the intersectionality of their disabilities and the other social determinants of health. Examples highlighted in other research include children with disabilities faced delays in returning to school when uncertainty about masking and physical distance protocols were not ascertained, elderly and those with dementia or intellectual disabilities and delays living in group home settings were more exposed to contamination and then faced more consequences of isolation on their mental and physical health, or neglect when there were not enough care workers for the daily healthcare management,^38^ lack of extra financial support for families of persons with disabilities who had to handle remote work or who had to continue working and provide direct care for people who have chronic conditions or require personal assistance, or persons with disabilities themselves who experienced financial hardship, job loss and delayed return to work due to lack of accommodations, particularly in low resources settings.^34,35,39,40^

 Though many countries considered at least in part the vulnerability of persons with disabilities in the public health services and measures, the maintenance and continuation of regular services, which are essential for the safeguards of rights, was less addressed. A World Health Organization (WHO) global report on disabilities and delays during the pandemic showed that the majority of children with disabilities in Canada did not receive adequate services to maintain their physical and mental health, did not receive adequate supports to maintain their education while their caregivers had to maintain work from home and other obligations.^41^ Similar challenges were reported by families of children with intellectual disabilities and ethnically diverse families in the United States.^42^ Studies on health services for diverse chronic conditions during the pandemic highlighted that a quick capacity to provide teleconsultations and reallocation of staff and facilities were some essential factors for service continuity and to avoid unintended long-term consequences that a lack of service could cause. Several guidelines and studies during the pandemic highlighted that the focus of health policies should not be solely on the frontline of contamination and immunization, but strengthen the community healthcare systems and adapt the structures so that patients with chronic healthcare conditions, heavy users of the system, could continue to receive services that were regarded as non-essential for the general population.^43^ There remains a need to harmonize and systematize approaches to addressing the unique needs of persons with disabilities in a pandemic or emergency context to make sure that human rights are respect in its integrity, and not limited to the access to facilities or to the emergency measures only.

 The need to systematize considerations for persons with disabilities includes the services beyond healthcare and across sectors. While some countries like India, South Africa and others noted the need to provide additional social and financial supports to carers and those living with disabilities, most of the policies focused on health protection and associated public health measures. The intersection of disability and other pre-existing factors contributing to vulnerability were seldom mentioned in policies. Increased risks for some sub-groups for social isolation, absence of health and rehabilitation services, basic food and income security, lack of access to real-time information and risk for abuse and neglect were highlighted by commentators^44^ and confirmed in early pandemic statistics.^11,45^ While the detailed provision suggested in the Articles of the UNCRPD on these matters was mentioned to some extent in policies protecting the right to access the community, education and health services, few countries addressed these elements in relation to protection from abuse, and considerations of isolation and social security in general. Article 16 of the convention states that States Parties shall take all appropriate legislative, administrative, social, educational and other measures to protect persons with disabilities, both within and outside the home, from all forms of exploitation, violence and abuse, including their gender-based aspects.^20^ Persons with disabilities were at increased risk of interpersonal violence and abuse^46^; although our model captured aspects in Article 23 that related mostly to people living in group homes, we did not identify particular protections from abuse and neglect. The cumulative impacts on physical and mental health are likely to continue and should be addressed in the following years’ policies and programs.

 Limitations in our study are to be noted. The use of the Cross-Industry Standard Process for Data Mining methodology is commonly employed for the analysis of large volumes of text data. It provides a numeric way to visualize the main information obtained in a corpus of documents and allows for a quick understanding of the text context.^47^ The development of a categorization model using the UNCRPD indicators is unique and more complex, allowing for identification of alignment between the language of the Convention and the policy documents, which we confirmed through several iterations of validation and a close read of the key words identified within the context of the full documents. However, this approach also has the limitation of reliance on the machine learning algorithm to initially capture and select the documents to be read closely. Our analysis is also limited by the low number of countries included, and restriction to English or French language documents, and the unequal inclusion of countries across income categories. We expect our purposeful sample can identify and illustrate issues pertaining to the condition of persons with disabilities across different countries, income categories and geographical locations, however we acknowledge that many of these details are only fully expressed at the sub-national levels of policies and programs, and much more is present in the lived experiences of individuals with disabilities during the pandemic.

 Attending to the rights of persons with disabilities in emergency responses and regular policy-making requires an intersectoral, multiple jurisdictions, comprehensive framework that considers the plurality of disabilities and the diverse responses needed. There are several potential uses of the UNCRPD as a model to guide future policies and practices that are more sensitive and responsive to different needs and to create a post-pandemic model of recovery that is more inclusive. Such efforts should consider the practical implications of policies, as well as the ability to include comprehensive and well-established frameworks so as to not neglect important aspects such as how people with disabilities (including subgroups like children, elderly and those living in institutions) will maintain their health and well-being, how children will maintain school services, and adults will maintain jobs and autonomy. The inclusion of these groups in close consultation during agenda setting and policy development will be crucial.

 Despite the fact that states are obliged to respect, protect, and fulfil disability rights in the context of a pandemic,^48^ our findings suggest that most states did not comply with human rights obligations owed to persons with disabilities during the COVID-19 pandemic. Our analysis also demonstrates that the UNCRPD’s potential role as a normative framework to develop equitable policy responses to health emergencies remains unfulfilled.^49^ Integrating the rights of persons with disabilities in public policy and programming contributes to the structural conditions that make pandemic response more coherent and responsive. Catching up during a pandemic is often unattainable for governments. For instance, having already established programs for income support, access to telehealth, inclusive transportation, and support for independent living will make individuals less vulnerable when an emergency situation occurs. Building a solid structure that follows normative frameworks such as the UNCRPD, and is applied effectively when an emergency strikes will support the return to an “improved normality” and stronger, person-centred, rights-promoting health systems.^50^

## Conclusion

 This analysis provides insights into the topics addressed by national level policies during the COVID-19 pandemic with particular emphasis on the rights of persons with disabilities. Our findings suggest that several countries addressed this population through various policy recommendations, but that these generally fell short of what is required under the UNCRPD. The overwhelming emphasis was on public health measures to contain and control the spread of COVID-19. In a few cases, there was important consideration given for the educational, financial, and other supports required by those living with disability. However, we find that less than half of our sample produced disability specific documents, and within these documents the policies were varied in the topics that they addressed. We find a continued need to establish comprehensive and standardized policy to address the wide-ranging rights of persons with disabilities in the context of a pandemic. In line with the obligations and principles set out in the UNCRPD, efforts to develop disability inclusive responses to future health emergencies require concrete mechanisms to involve persons with disabilities in decision-making processes and dedicated, proactive policy measures to safeguard their fundamental human rights to life, health, education, work, standard of living, and community inclusion.

## Acknowledgements

 The team would like to thank the ongoing support of stakeholder partners. A warm thank you to Steve Estey (independent disability advocate consultant), Kerri Joffe (ARCH Disability Law Clinic), Neil Belanger (British Columbia Aboriginal Disability Network, National COVID-19 Disability Advisory), Krista Carr (Inclusion Canada), Kathy Vandergrift (Canadian Coalition for the Rights of Children), and Nicholas Katalifo (McGill University Montreal Neurological Institute, Azrieli Centre for Autism Research, Disability Parent Advocate).

## Ethical issues

 No ethics review was required for the project, no human subjects were recruited as participants.

## Competing interests

 Authors declare that they have no competing interests.

## Funding

 The project was funded by the Réseau de recherche en santé des populations du Québec (RRSPQ); Réseau provincial de recherche en adaptation-réadaptation (REPAR). KS work is supported by the Canada Research Chairs program, MH, AH are supported by a Chercheur Boussier funds from the Fonds de Recherche du Québec-Santé. KS and SJ work are supported by the Canada Research Chairs Program.

## Supplementary files


Supplementary file 1. Policy Collection Sources.

Supplementary file 2. Categorization Model Matrix Example.

Supplementary file 3. Most Frequently Occurring Phrases in All Documents (Excluding Duplicate or Republished Documents) for All 14 Countries Over the Collection Period.

Supplementary file 4. Topic Extraction Conducted Using WordStat of All Documents (Excluding Duplicate or Republished Documents) Collected Over the 12-month Period for All 14 Countries.

Supplementary file 5. Cooccurrence Analysis Conducted Using WordStat of All Documents (Excluding Duplicate or Republished Documents) for All 14 Countries Over the Collection Period.
