# Supplementary material for: How Did Governments Address the Needs of People With Disabilities During the COVID-19 Pandemic? An Analysis of 14 Countries’ Policies Based on the UN Convention on the Rights of Persons With Disabilities
Source: Int J Health Policy Manag. 2023 May 17;12:7111. doi: 10.34172/ijhpm.2023.7111 (PMC10425656; doi:10.34172/ijhpm.2023.7111)
Supplement: Supplementary file 1 — Policy Collection Sources. [file ijhpm-12-7111-s001.pdf]

**Article title:** How Did Governments Address the Needs of People With Disabilities During the COVID-19 Pandemic? An Analysis of 14 Countries' Policies Based on the UN Convention on the Rights of Persons With Disabilities

**Journal name:** International Journal of Health Policy and Management (IJHPM)

**Authors' information:** Keiko Shikako[1,2]*, Raphael Lencucha[1,2], Matthew Hunt[1,2], Sébastien Jodoin[3], Mayada Elsabbagh[4], Anne Hudon[5], Derrick Cogburn[6,7], Ananya Chandra[1,2], Anna Gignac-Eddy[3], Nilani Ananthamoorthy[3], Rachel Martens[8]

[1]School of Physical and Occupational Therapy, McGill University, Montreal, QC, Canada.
[2]Center for Interdisciplinaire Research in Rehabilitation of the Greater Montreal (CRIR), Montreal, QC, Canada.
[3]Faculty of Law, McGill University, Montreal, QC, Canada.
[4]Montreal Neurological Institute, McGill University, Montreal, QC, Canada.
[5]School of Rehabiltiation, University of Montreal, Montreal, QC, Canada.
[6]School of International Service and Kogod School of Business, American University, Washington, DC, USA.
[7]Institute on Disability and Public Policy (IDPP), American University, Washington, DC, USA.
[8]Kids Brain Health Network, CanChild, Calgary, AB, Canada.

(*Corresponding author: keiko.thomas@mcgill.ca)

**Supplementary file 1.** Policy Collection Sources

| Country | Source(s) |
|---|---|
| Canada | Public Health Agency of Canada's Coronavirus online focal point |
| India | Ministry of Health |
| | Department of Empowerment of Persons with Disabilities (Ministry of Social Justice and Empowerment) |
| Australia | Communicable Diseases Network Australia |
| Ireland | Department of Health |
| Jamaica | Jamaica Information System |
| | Jamaica Council for Persons with Disabilities |
| Fiji | Ministry of Health and Medical Services |
| | Fiji.gov COVID online focal point |
| South Africa | National Disaster Management Center |
| | (Department Of Co-Operative Governance and Traditional Affairs) |
| | Gov.za COVID online focal point |

| | |
|---|---|
| France | Groupement National Centres Ressources Autisme |
| | Legifrance |
| | La Directrice de l'information légale et administrative |
| | Ministère Des Solidarités De L'autonomie Et Des Personnes Handicapées |
| Zimbabwe | Government Gazette |
| | Ministry of Health and ChildCare |
| Rwanda | Prime Minister's Office |
| | Ministry of Health |
| Philippines | Center for Disaster Preparedness |
| | President's Office |
| | Department of Social Welfare and Development |
| | Department of Health |
| Guinea | Republic of Guinea government website |
| Haiti | Ministere De La Sante Publique Et De La Population |
| Malawi | Government Gazette |
| | Ministry of Health |
| | Ministry of Disaster Management Affairs and Public Events |
