# Supplementary material for: How Did Governments Address the Needs of People With Disabilities During the COVID-19 Pandemic? An Analysis of 14 Countries’ Policies Based on the UN Convention on the Rights of Persons With Disabilities
Source: Int J Health Policy Manag. 2023 May 17;12:7111. doi: 10.34172/ijhpm.2023.7111 (PMC10425656; doi:10.34172/ijhpm.2023.7111)
Supplement: Supplementary file 2 — Categorization Model Matrix Example. [file ijhpm-12-7111-s002.pdf]

Article title: How Did Governments Address the Needs of People With Disabilities During the COVID-19 Pandemic? An Analysis of 14 Countries' Policies Based on the UN Convention on the Rights of Persons With Disabilities

Journal name: International Journal of Health Policy and Management (IJHPM)

Authors' information: Keiko Shikako[1,2]*, Raphael Lencucha[1,2], Matthew Hunt[1,2], Sébastien Jodoin[3], Mayada Elsabbagh[4], Anne Hudon[5], Derrick Cogburn[6,7], Ananya Chandra[1,2], Anna Gignac-Eddy[3], Nilani Ananthamoorthy[3], Rachel Martens[8]

[1]School of Physical and Occupational Therapy, McGill University, Montreal, QC, Canada.

[2]Center for Interdisciplinaire Research in Rehabilitation of the Greater Montreal (CRIR), Montreal, QC, Canada.

[3]Faculty of Law, McGill University, Montreal, QC, Canada.

[4]Montreal Neurological Institute, McGill University, Montreal, QC, Canada.

[5]School of Rehabiltiation, University of Montreal, Montreal, QC, Canada.

[6]School of International Service and Kogod School of Business, American University, Washington, DC, USA.

[7]Institute on Disability and Public Policy (IDPP), American University, Washington, DC, USA.

[8]Kids Brain Health Network, CanChild, Calgary, AB, Canada.

(*Corresponding author: keiko.thomas@mcgill.ca)

Supplementary file 2. Categorization Model Matrix Example[1]

**Article**	**Example**<br>**Subcategory: STRUCTURE**
Article 11<br><br>Attributes:<br>1. **Prevention and Preparedness**<br>2. **Rescue and Response**<br>3. **Recovery, Reconstruction, and Reconciliation**	**Indicator**: 11.4 Adoption of legal requirement to ensure the inclusion and participation of persons with disabilities in humanitarian needs assessments, related monitoring processes, and in all programmes and projects related to situations of risk and humanitarian emergencies, climate-related hazards and disaster risk reduction.
	**Rule**: [DISAB* AND DISASTER_RISK_REDUC* AND HUMAN*_EMERGEN* AND HUMAN*_NEED*_ASSESSMENT AND CLIMATE*-RELATED_HAZARD* /Y /S/S/S/S] (1)
	**Quote**: "the TG disability Disaster Risk Reduction Covid19 group launched a survey on disabilities and COVID19 to have

	data and to help governments to have inclusive Covid19 response" (South Africa)
Article 24<br><br>Attributes:<br>4. **Inclusive education system**<br>5. **Quality and free primary and secondary education**<br>6. **Access to tertiary, vocational training and lifelong learning**<br>7. **Inclusive teaching**	**Indicator**: 24.9 Legislation enacted that prohibits violence, corporal punishment, bullying and harassment in educational environments including on the basis of disability.
	**Rule**: [VIOLEN* AND LEG* AND SCHOOL* AND DISAB* /A/D/P/P] (1)
	**Quote**: "School mitigation measures such as closures have a high economic and social cost. Schools and childcare facilities play many important roles in communities. They provide necessary education to prepare children for adulthood, and long term online education for K-12 is not a substitute for in-person learning and socialization in a school setting. Long-term shutdowns have the potential to lead to education gaps and other consequences for many children. Particular groups may be disproportionately affected, such as children who receive school-based meals or counselling, children who are in situations of domestic violence or abuse, children with disabilities who rely on the certainty of education and social supports to maintain health and community inclusion, and essential workers from low income families or single parents who have not had access to flexible work arrangements or paid leave. As businesses reopen, many workers will be dependent on access to child care, schools and child care facilities." (Canada)
Article 19<br><br>Attributes:<br>1. **Choice of independent living arrangements**<br>2. **Support services**<br>3. **Accessibility and responsiveness of mainstream services**	**Indicator**: 19.10 Availability of support measures, including home support, peercounselling and financial support or allowance for persons with disabilities and those relatives and/or others with whom the person decides to live.
	**Rule**: [DISAB* AND SUPPORT* AND COUNSEL* AND FINANCIAL_SUPPORT* AND ALLOWANCE* /Y /S/S/S/S] (1)
	**Quote**: "If a disability service provider or support worker refuses to continue providing supports to you because you decided not to get the COVID-19 vaccine, it could be a breach of the NDIS Code of Conduct (NDIS Providers). In this case, a complaint can be made to the NDIS Commission."

[1]Articles selected were the ones with more frequent citations in this study
