# Supplementary material for: How Did Governments Address the Needs of People With Disabilities During the COVID-19 Pandemic? An Analysis of 14 Countries’ Policies Based on the UN Convention on the Rights of Persons With Disabilities
Source: Int J Health Policy Manag. 2023 May 17;12:7111. doi: 10.34172/ijhpm.2023.7111 (PMC10425656; doi:10.34172/ijhpm.2023.7111)
Supplement: Supplementary file 3 — Most Frequently Occurring Phrases in All Documents (Excluding Duplicate or Republished Documents) for All 14 Countries Over the Collection Period. [file ijhpm-12-7111-s003.pdf]

Article title: How Did Governments Address the Needs of People With Disabilities During the COVID-19 Pandemic? An Analysis of 14 Countries' Policies Based on the UN Convention on the Rights of Persons With Disabilities

Journal name: International Journal of Health Policy and Management (IJHPM)

Authors' information: Keiko Shikako[1,2]*, Raphael Lencucha[1,2], Matthew Hunt[1,2], Sébastien Jodoin[3], Mayada Elsabbagh[4], Anne Hudon[5], Derrick Cogburn[6,7], Ananya Chandra[1,2], Anna Gignac-Eddy[3], Nilani Ananthamoorthy[3], Rachel Martens[8]

[1]School of Physical and Occupational Therapy, McGill University, Montreal, QC, Canada.

[2]Center for Interdisciplinaire Research in Rehabilitation of the Greater Montreal (CRIR), Montreal, QC, Canada.

[3]Faculty of Law, McGill University, Montreal, QC, Canada.

[4]Montreal Neurological Institute, McGill University, Montreal, QC, Canada.

[5]School of Rehabiltiation, University of Montreal, Montreal, QC, Canada.

[6]School of International Service and Kogod School of Business, American University, Washington, DC, USA.

[7]Institute on Disability and Public Policy (IDPP), American University, Washington, DC, USA.

[8]Kids Brain Health Network, CanChild, Calgary, AB, Canada.

(*Corresponding author: keiko.thomas@mcgill.ca)

Supplementary file 3. Most Frequently Occurring Phrases in All Documents (Excluding Duplicate or Republished Documents) for All 14 Countries Over the Collection Period

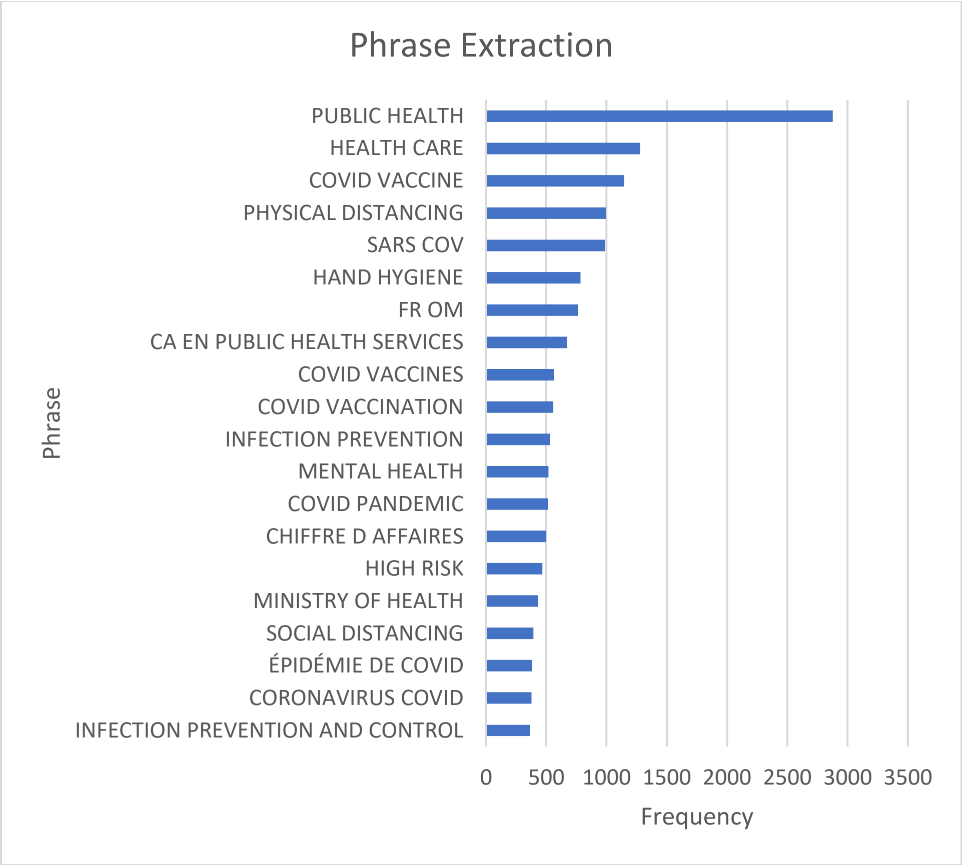
Phrase Extraction
PUBLIC HEALTH
HEALTH CARE
COVID VACCINE
PHYSICAL DISTANCING
SARS COV
HAND HYGIENE
FR OM
CA EN PUBLIC HEALTH SERVICES
COVID VACCINES
COVID VACCINATION
INFECTION PREVENTION
MENTAL HEALTH
COVID PANDEMIC
CHIFFRE D AFFAIRES
HIGH RISK
MINISTRY OF HEALTH
SOCIAL DISTANCING
ÉPIDÉMIE DE COVID
CORONAVIRUS COVID
INFECTION PREVENTION AND CONTROL
Phrase
0 500 1000 1500 2000 2500 3000 3500
Frequency
