# Supplementary material for: How Did Governments Address the Needs of People With Disabilities During the COVID-19 Pandemic? An Analysis of 14 Countries’ Policies Based on the UN Convention on the Rights of Persons With Disabilities
Source: Int J Health Policy Manag. 2023 May 17;12:7111. doi: 10.34172/ijhpm.2023.7111 (PMC10425656; doi:10.34172/ijhpm.2023.7111)
Supplement: Supplementary file 4 — Topic Extraction Conducted Using WordStat of All Documents (Excluding Duplicate or Republished Documents) Collected Over the 12-month Period for All 14 Countries. [file ijhpm-12-7111-s004.pdf]

Article title: How Did Governments Address the Needs of People With Disabilities During the COVID-19 Pandemic? An Analysis of 14 Countries' Policies Based on the UN Convention on the Rights of Persons With Disabilities

Journal name: International Journal of Health Policy and Management (IJHPM)

Authors' information: Keiko Shikako[1,2]*, Raphael Lencucha[1,2], Matthew Hunt[1,2], Sébastien Jodoin[3], Mayada Elsabbagh[4], Anne Hudon[5], Derrick Cogburn[6,7], Ananya Chandra[1,2], Anna Gignac-Eddy[3], Nilani Ananthamoorthy[3], Rachel Martens[8]

[1]School of Physical and Occupational Therapy, McGill University, Montreal, QC, Canada.
[2]Center for Interdisciplinaire Research in Rehabilitation of the Greater Montreal (CRIR), Montreal, QC, Canada.
[3]Faculty of Law, McGill University, Montreal, QC, Canada.
[4]Montreal Neurological Institute, McGill University, Montreal, QC, Canada.
[5]School of Rehabiltiation, University of Montreal, Montreal, QC, Canada.
[6]School of International Service and Kogod School of Business, American University, Washington, DC, USA.
[7]Institute on Disability and Public Policy (IDPP), American University, Washington, DC, USA.
[8]Kids Brain Health Network, CanChild, Calgary, AB, Canada.

(*Corresponding author: keiko.thomas@mcgill.ca)

Supplementary file 4. Topic Extraction Conducted Using WordStat of All Documents (Excluding Duplicate or Republished Documents) Collected Over the 12-month Period for All 14 Countries

**Topic**	**Keywords**	**Freq**	**Cases**	**% cases**
Transmission	from; transmission; pr actices; pr o vide; pre vention; required; appropriate; spread; provincial; pr e vent; control; progr; precautions;	8082	105	13.74%
Présent Article mentionnés	Article; mentionnés; code; établissements; présent; peuvent; dispositions; application; ii; santé; décret;<br><br>présent Article; présent décret; santé publique; code de la santé publique; notamment son Article; établissements mentionnés; conditions prévues;	5551	334	43.72%

Perte de chiffre affaires de référence	Chiffre; affaires; entreprises; mois; période; mars;<br><br>perte de chiffre; affaires de référence; subi une perte de chiffre; période comprise; affaires réalisé; entreprises mentionnées; affaires mensuel; deux mois; période éligible; affaires dans la limite; entreprises du secteur; entreprises créées; cours du mois; affaires de référence mentionné; affaires est réalisé; titre du mois; affaires mensuel moyen; date de création; entreprises bénéficient; coûts fixes; iv du présent Article; activités de vente; période semestrielle; montant de la subvention; fonds de solidarité; exploitation coûts fixes; mois de février; accueil du public; résultat net; excédent brut;	1950	112	14.66%
Based hand	Hands; wash; alcohol; hand; sanitize; water;<br><br>soap and water; based hand; wash hands; hand sanitizer; hand washing; sanitize or wash; alcohol or above; based hand sanitizer; hand sanitizers; wash your hands; based hand rub; hands with soap and water; hands are not visibly; sanitize or wash hands; hand wash; alcohol based;	3804	263	34.42%
Key populations vaccine supply	immunization; vaccine; populations; vaccines; recommendations; evidence; key;<br><br>key populations; vaccine supply; health services immunization national;	5151	189	24.74%
Acute respiratory	Respiratory; acute; severe; illness; fever; symptoms; signs;<br><br>acute respiratory; severe acute respiratory; respiratory illness; respiratory distress; signs and symptoms; respiratory symptoms; severe acute respiratory syndrome; acute respiratory illness; acute respiratory infection; acute respiratory distress; respiratory infections; respiratory tract; acute respiratory distress syndrome;	3967	318	41.62%
State government	State; district; authority; government; national; disaster;<br><br>disaster management; state government; state and district; state of disaster; national state of disaster; national authority; state authority; district level; central government; state and territory; australian government; district authority; district helpline;	4346	418	54.71%
Confirmed cases	Confirmed; case; cases; contacts; isolation; suspected; contact; quarantine;<br><br>confirmed case; confirmed cases; confirmed covid; contact tracing; suspected or confirmed; close contacts; cases of covid; close contact; suspected cases;	6001	425	55.63%

Personal protective equipment	Equipment; personal; ppe; hygiene; hand;<br><br>personal protective; personal protective equipment; hand hygiene; personal pr; personal hygiene; personal pr otective; personal pr otective equipment; perform hand hygiene;	4081	305	39.92%
Coronavirus	Onavirus; cor; vid;<br><br>coronavirus; coronavirus disease; coronavirus; ca coronavirus disease;	1424	58	7.59%
Respiratory	respiratory; acute respiratory; espiratory etiquette; droplets; respiratory symptoms; droplets; sneezing; syndrome; distress; laboratory;	669	36	4.71%
Response plan preparedness	Preparedness; response; plan;<br><br>response plan; preparedness and response plan; emergency response; national covid; emergency response plan; response activities; australian health sector;	2093	235	30.76%
Premier ministre alinéa	Premier; alinéa; ministre; mots;<br><br>premier ministre; premier alinéa; remplacés par les mots; auprès du ministre; finances et de la relance; ministre délégué auprès du ministre; exécution du présent décret; chargé des comptes publics; insérés les mots; sera publié; solidarités et de la santé; troisième alinéa; alinéa ainsi rédigé;	1872	151	19.76%
Cleaning and disinfection touch surfaces	Cleaning; surfaces; disinfection; clean;<br><br>cleaning and disinfection; touch surfaces; touched surfaces; envir onmental cleaning; envir onmental; frequently touched surfaces; environmental cleaning; cleaning and disinfecting;	2899	217	28.40%
Infection prevention control	Control; prevention; infection;<br><br>infection prevention; infection prevention and control; infection control; control measures; control practices; disease control;	4559	344	45.03%
Face mask	Face; mask; masks;<br><br>face mask; face masks; cloth face; face covers; medical masks; face cover; face to face; face covers masks; face shield; medical mask; limited face; face classes; face co vering;	5061	335	43.85%
Sars cov	Sars; cov;	2172	182	23.82%
Years of age	Age; years; dose;<br><br>years of age; age groups; after dose;	1899	202	26.44%

Situation de handicap	Handicap; situation; personnes; mesures;<br><br>situation de handicap; personnes en situation de handicap; personnes handicapées; personnes morales; personnes physiques; dirigeant majoritaire; personnes accueillies;	1558	180	23.56%
Telemedicine consultation	Rmp; telemedicine; consultation; patient;<br><br>telemedicine consultation;	2122	190	24.87%
High risk transmission	Risk; transmission; high;<br><br>high risk; risk of transmission; risk assessment; risk factors; higher risk; risk of severe; risk mitigation;	3790	358	46.86%
Health act disaster management	Act; section; regulations; disaster;<br><br>health act; disaster management; terms of section; disaster management act; temporary restrictions; liquor act;	2969	246	32.20%
Physical distancing	Distancing; physical; social;<br><br>physical distancing; social distancing; physical distancing norms; social distancing measures; maintain physical distancing;	4286	322	42.15%
Health care essential	Essential; workers; health;<br><br>health care; public health; health services; health workers; health care workers; news health; alert coronavirus; mental health; health facilities; essential service; essential services; infection health;	5776	469	61.39%
Term care long	Long; term; care;<br><br>term care; long term; care facilities; term care facilities; documents residents; long term care;	2663	261	34.16%
