# Supplementary material for: How Did Governments Address the Needs of People With Disabilities During the COVID-19 Pandemic? An Analysis of 14 Countries’ Policies Based on the UN Convention on the Rights of Persons With Disabilities
Source: Int J Health Policy Manag. 2023 May 17;12:7111. doi: 10.34172/ijhpm.2023.7111 (PMC10425656; doi:10.34172/ijhpm.2023.7111)
Supplement: Supplementary file 5 — Cooccurrence Analysis Conducted Using WordStat of All Documents (Excluding Duplicate or Republished Documents) for All 14 Countries Over the Collection Period. [file ijhpm-12-7111-s005.pdf]

Article title: How Did Governments Address the Needs of People With Disabilities During the COVID-19 Pandemic? An Analysis of 14 Countries' Policies Based on the UN Convention on the Rights of Persons With Disabilities

Journal name: International Journal of Health Policy and Management (IJHPM)

Authors' information: Keiko Shikako[1,2]*, Raphael Lencucha[1,2], Matthew Hunt[1,2], Sébastien Jodoin[3], Mayada Elsabbagh[4], Anne Hudon[5], Derrick Cogburn[6,7], Ananya Chandra[1,2], Anna Gignac-Eddy[3], Nilani Ananthamoorthy[3], Rachel Martens[8]

[1]School of Physical and Occupational Therapy, McGill University, Montreal, QC, Canada.
[2]Center for Interdisciplinaire Research in Rehabilitation of the Greater Montreal (CRIR), Montreal, QC, Canada.
[3]Faculty of Law, McGill University, Montreal, QC, Canada.
[4]Montreal Neurological Institute, McGill University, Montreal, QC, Canada.
[5]School of Rehabiltiation, University of Montreal, Montreal, QC, Canada.
[6]School of International Service and Kogod School of Business, American University, Washington, DC, USA.
[7]Institute on Disability and Public Policy (IDPP), American University, Washington, DC, USA.
[8]Kids Brain Health Network, CanChild, Calgary, AB, Canada.

(*Corresponding author: keiko.thomas@mcgill.ca)

Supplementary file 5. Cooccurrence Analysis Conducted Using WordStat of All Documents (Excluding Duplicate or Republished Documents) for All 14 Countries Over the Collection Period

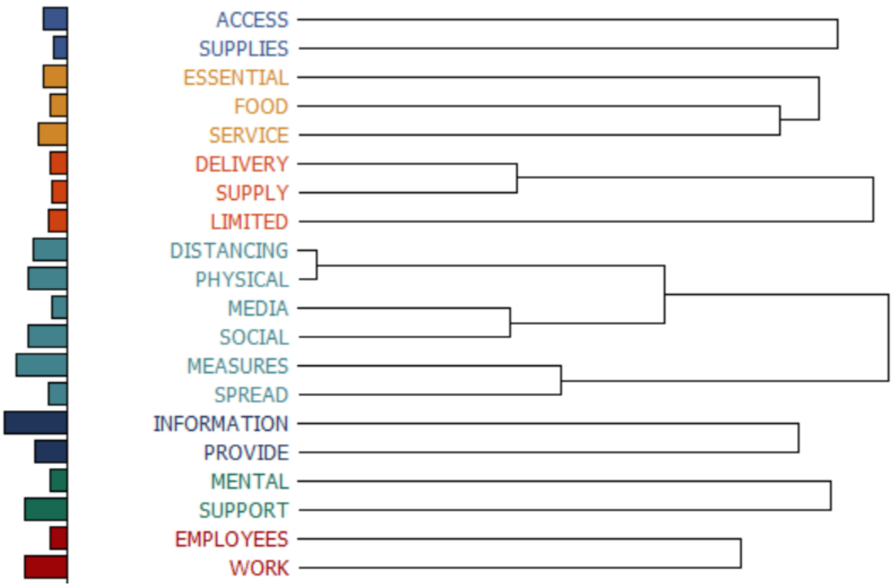
ACCESS
SUPPLIES
ESSENTIAL
FOOD
SERVICE
DELIVERY
SUPPLY
LIMITED
DISTANCING
PHYSICAL
MEDIA
SOCIAL
MEASURES
SPREAD
INFORMATION
PROVIDE
MENTAL
SUPPORT
EMPLOYEES
WORK

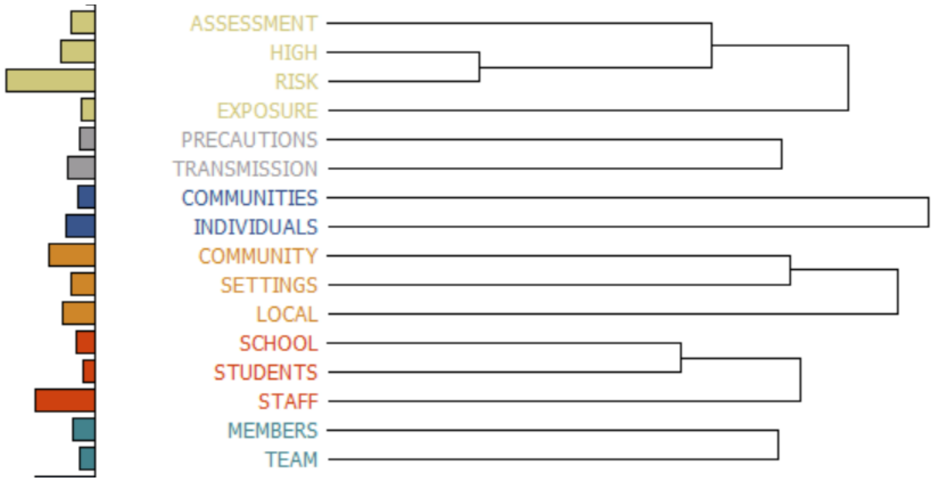
ASSESSMENT
HIGH
RISK
EXPOSURE
PRECAUTIONS
TRANSMISSION
COMMUNITIES
INDIVIDUALS
COMMUNITY
SETTINGS
LOCAL
SCHOOL
STUDENTS
STAFF
MEMBERS
TEAM
